# A holistic view of cancer bioenergetics: mitochondrial function and respiration play fundamental roles in the development and progression of diverse tumors

**DOI:** 10.1186/s40169-016-0082-9

**Published:** 2016-01-26

**Authors:** Md Maksudul Alam, Sneha Lal, Keely E. FitzGerald, Li Zhang

**Affiliations:** Department of Biological Sciences, University of Texas at Dallas, Mail Stop RL11, 800 W, Campbell Road, Richardson, TX 75080 USA

**Keywords:** Tumor bioenergetics, Glutamine, Tumor heterogeneity, Metabolic mutations, Mitochondrial respiration, Oxidative phosphorylation, Heme, Hemoprotein, Mitochondrial transfer

## Abstract

Since Otto Warburg made the first observation that tumor cells exhibit altered metabolism and bioenergetics in the 1920s, many scientists have tried to further the understanding of tumor bioenergetics. Particularly, in the past decade, the application of the state-of the-art metabolomics and genomics technologies has revealed the remarkable plasticity of tumor metabolism and bioenergetics. Firstly, a wide array of tumor cells have been shown to be able to use not only glucose, but also glutamine for generating cellular energy, reducing power, and metabolic building blocks for biosynthesis. Secondly, many types of cancer cells generate most of their cellular energy via mitochondrial respiration and oxidative phosphorylation. Glutamine is the preferred substrate for oxidative phosphorylation in tumor cells. Thirdly, tumor cells exhibit remarkable versatility in using bioenergetics substrates. Notably, tumor cells can use metabolic substrates donated by stromal cells for cellular energy generation via oxidative phosphorylation. Further, it has been shown that mitochondrial transfer is a critical mechanism for tumor cells with defective mitochondria to restore oxidative phosphorylation. The restoration is necessary for tumor cells to gain tumorigenic and metastatic potential. It is also worth noting that heme is essential for the biogenesis and proper functioning of mitochondrial respiratory chain complexes. Hence, it is not surprising that recent experimental data showed that heme flux and function are elevated in non-small cell lung cancer (NSCLC) cells and that elevated heme function promotes intensified oxygen consumption, thereby fueling tumor cell proliferation and function. Finally, emerging evidence increasingly suggests that clonal evolution and tumor genetic heterogeneity contribute to bioenergetic versatility of tumor cells, as well as tumor recurrence and drug resistance. Although mutations are found only in several metabolic enzymes in tumors, diverse mutations in signaling pathways and networks can cause changes in the expression and activity of metabolic enzymes, which likely enable tumor cells to gain their bioenergetic versatility. A better understanding of tumor bioenergetics should provide a more holistic approach to investigate cancer biology and therapeutics. This review therefore attempts to comprehensively consider and summarize the experimental data supporting our latest view of cancer bioenergetics.

## Introduction

Terrestrial organisms vary in many ways, but one characteristic common to all living organisms is the need for cellular energy. The universal energy currency is ATP. Eukaryotes ranging from yeast to humans generate ATP via glycolysis and oxidative phosphorylation. The term glycolysis comes from the Greek “glyco-,” meaning “sweet,” and “-lysis,” meaning “to split.” The name is apt, as the glycolytic pathway involves the splitting of sugar to produce ATP. Oxidative phosphorylation (OXPHOS) is so named because it combines inorganic phosphate with ADP to form ATP in the presence of oxygen. Human cells can use various fuels, including glucose, amino acids, and fat for ATP production to support cellular function and proliferation. Fuel usage for ATP generation is dependent on the conditions of the body. For example, in healthy, well-fed individuals, skeletal muscle is degraded and regenerated frequently. The amino acid pool in humans remains relatively unchanged. To keep the amino acid pool constant during starvation, the degradation of skeletal muscles increases. This occurs so that the body can continue to provide energy for essential functions. Glutamine and alanine constitute the majority of amino acids released from skeletal muscles during starvation. One consequence of unlimited cancer cell proliferation is likely to make the human body feel starved and respond in a way similar to the body’s response to starvation. Hence, it is conceivable that glutamine can be a preferred fuel for many types of cancer cells. The importance of glutamine in tumor metabolism and bioenergetics is further confirmed by recent metabolomics studies showing that α-ketoglutarate from glutamine can undergo reductive carboxylation to generate citrate, which can be turned into malate for generating NADPH via malic enzyme. This provides an alternative pathway for cancer cells to generate citrate and NADPH for sustaining anabolic reactions. Another noteworthy development in cancer bioenergetics research is the finding that stromal cells in the tumor microenvironment can provide cancer cells with bioenergetic substrates. Below, we consider previous and emerging research results and attempt to provide a holistic and up-to-date view of tumor bioenergetics.

## Review

### High glycolytic rates occur concomitantly with oxidative phosphorylation (OXPHOS) in cells of most tumors

**Fig. 1:**
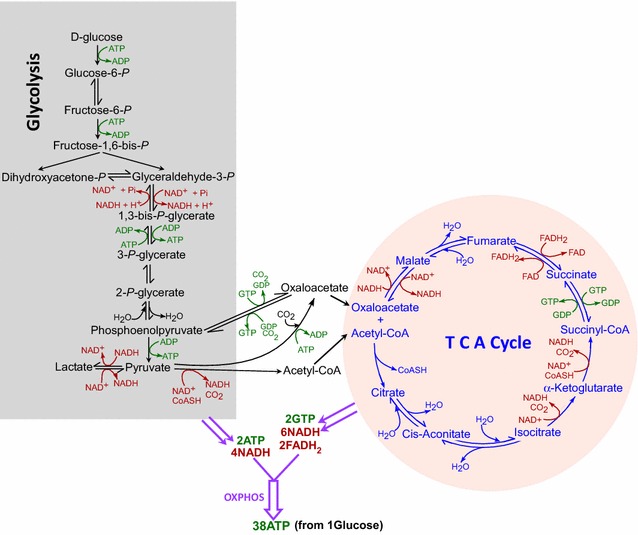
The metabolic steps of glycolysis and TCA cycle. The steps involved in glycolysis and TCA cycle are demarcated separately. ATP/GTP utilization or synthesis is shown in green, while NAD^+^/NADH and FAD/FADH_2_ are shown in red. Also indicated are the numbers of NADH, FADH_2_, and ATP/GTP generated when one molecule of glucose is consumed following glycolysis, TCA cycle, and oxidative phosphorylation. Abbreviations: *Glucose*-*6*-*P* glucose 6-phosphate, *Fructose*-*6*-*P* fructose 6-phosphate, *Fructose* -*1,6*-*bis*-*P* fructose 1,6-bisphosphate, *Dihydroxyacetone*-*P* dihydroxyacetone phosphate, *Glyceraldehyde*-*3*-*P* glyceraldehyde 3-phosphate, *1,3*-*Bis*-*P*-*glycerate* 1,3-bisphosphoglycerate, *3*-*P*-*Glycerate* 3-phosphoglycerate, *2*-*P*-*Glycerate* 2-phosphoglycerate, *OXPHOS* oxidative phosphorylation

Glycolysis was first studied by Louis Pasteur in an attempt to understand the process of fermentation in 1856 [1]. Glycolysis as we understand it today was finalized by Buchner in 1947 [2]. Glycolysis consumes 2 ATP and produces 4 ATP for a net yield of 2 ATP (Fig. 1). In the absence of oxygen, glycolysis is the metabolic pathway of choice. In the presence of oxygen, however, glycolysis only begins the process of aerobic respiration. In the presence of oxygen, pyruvate is consumed by the tricarboxylic acid (TCA) cycle (Fig. 1). Albert Szent-Gyorgyi made major contributions elucidating the TCA cycle, also known as the Krebs cycle, in the 1920s and 1930s [3–7]. In 1945, coenzyme A was discovered by Fritz Lipmann [8]. However, the most important and well-known contributor to the discovery and understanding of the TCA cycle is Hans Krebs, who discovered that cycle began with citrate [9]. The TCA cycle does not produce ATP directly, although it produces 1 GTP, which is easily converted to ATP (Fig. 1). However, the TCA cycle is extremely important for energy production because it provides the precursor molecules, namely NADH and FADH_2_, for OXPHOS (Fig. 1). Breakthroughs in OXPHOS were made from 1944 to 1980, beginning with Dickens, McIlwain, Neuberger, Norris, Obrien, and Young and ending with Boyer [10–12]. OXPHOS is the preferred energy-generating method of many life forms, including mammals, in the presence of oxygen. This is because OXPHOS creates up to 38 ATP molecules per one molecule of glucose, as compared to only 2 ATP molecules generated anaerobically by glycolysis (Fig. 1). Both TCA cycle and OXPHOS occur in mitochondria.

How cancer cells gain sufficient ATP to support their unabated proliferation and function has fascinated many scientists for nearly a century. The German scientist Otto Warburg and co-workers performed the first quantitative study of cancer cell metabolism in the 1920s [13, 14]. They showed that tumor tissues metabolize approximately tenfold more glucose to lactate in a given time than normal tissues, even when presented with enough oxygen to metabolize glucose completely to CO_2_. This phenomenon is widely known as the Warburg effect and is the origin of the perception that a high glycolytic rate is typical of cancer/tumor cells [15]. The rationale for the high glycolytic rate was that tumor mitochondria have impaired respiration, which is compensated by an unusually high contribution of aerobic glycolysis to sustain ATP generation. The hallmark of aerobic glycolysis is a high rate of lactate production from glucose in the presence of oxygen. Warburg’s observation has motivated generations of cancer biologists and biochemists to refine his hypothesis and provide mechanistic explanations for it.

However, immediately after the publication of Warburg’s paper “On the Origin of Cancer Cells” [15], Weinhouse contested Warburg’s ideas based on results in his laboratory showing that neoplastic tissues have a normal OXPHOS (oxidative phosphorylation) capacity when supplemented with NAD^+^ [16, 17]. In 1979, Reitzer and co-workers showed that in cultured HeLa cells, more than half of the ATP requirement (determined by comparing ^14^CO_2_ production from ^14^C-glutamine and ^14^C-lactate production from ^14^C-glucose) comes from glutamine even when a high concentration (10 mM) of glucose is present [18]. When fructose or galactose is the carbohydrate, glutamine provides greater than 98 % of energy by aerobic oxidation from the TCA cycle. Experimental studies in recent years have confirmed the idea that glutamine is a major nutrient in cancer cells [19–22]. Additionally, ample experimental evidence showed that glutamine is a good substrate for oxidative metabolism in various tumor and cancer cells [23–26]. It is also worth noting that the authors’ lab recently showed that glutamine enables an array of non-small cell lung cancer (NSCLC) cells to increase oxygen consumption substantially when glucose is depleted [27]. Taken together, various studies have shown that glucose and glutamine are key nutrients and fuels for cancer cells [21, 28]. Different cancer cells may exhibit varying dependence on glucose or glutamine [29, 30].

### Both glucose and glutamine are important nutrients for many types of cancer cells and tumors

**Fig. 2:**
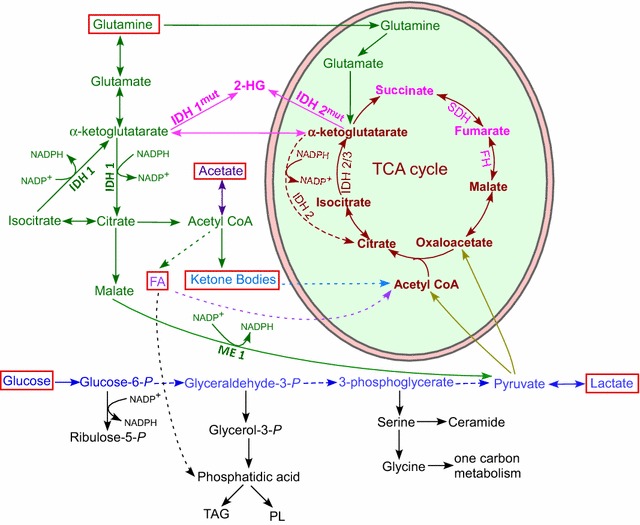
Metabolic fuels for tumor cells. Tumor cells are able to use a variety of bioenergetic substrates, including glutamine, glucose, fatty acids (FA), ketone bodies, and acetate (highlighted by *red boxes*). These substrates can be provided by stromal cells in the microenvironment. Much of the cellular energy for tumor cells is likely generated by TCA cycle coupled to oxidative phosphorylation. The pathways for the generation and metabolism of these substrates are outlined. Notably, glutamine and glucose can also provide building blocks for the synthesis of many biomolecules. Also indicated in pink are the metabolic enzymes whose mutations are found in various tumors and the accumulated oncometabolites in these tumors

The importance of glutamine as a nutrient and fuel is consistent with the fact it is the most abundant amino acid released from skeletal muscle, and it is the most abundant amino acid in plasma [31]. The importance of glucose and glutamine in cancer metabolism and bioenergetics can be easily gleaned from the architecture of metabolic pathways (Fig. 2). Both glucose and glutamine have dual roles in ATP production and biosynthesis (anabolism). Although glucose can generate ATP via glycolysis, its most prominent function is evidently in anabolism (biosynthesis). As shown in Fig. 2, glucose can generate the precursor ribulose-5-P and the reducing power NADPH via the pentose phosphate pathway. The glycolysis intermediate glyceraldehyde 3-P can yield glycerol-3-P, which serves as a backbone for the synthesis of phosphatidic acid, a precursor for the synthesis of triacylglycerol and phospholipids. Another glycolysis intermediate, 3-P glycerate, is a precursor for serine, which can be used to synthesize ceramide and is a precursor for one-carbon metabolism. Ultimately, pyruvate generated from glucose via glycolysis can be turned into Acetyl CoA and serves as a substrate feeding the TCA cycle. Under conditions permitting mitochondrial respiration, NADH and FADH_2_ generated from the TCA cycle will lead to high yield of ATP, via the electron transport chain and OXPHOS (Fig. 1).

Glutamine is a very versatile nutrient feeding into many pathways for ATP generation, redox homeostasis, and biosynthesis [22]. Firstly, glutamine is the main substrate supporting TCA cycle anaplerosis. Glutamine can be readily turned into α-ketoglutarate, which feeds the TCA cycle (Fig. 2), leading to the generation of NADH and FADH_2_, which is used to generate ATP via electron transport and OXPHOS (Fig. 1). This can lead to the generation of various TCA cycle intermediates, which can support many biosynthetic reactions and gluconeogenesis. Secondly, under hypoxic conditions or when mitochondria are defective, α-ketoglutarate from glutamine can undergo reductive carboxylation to generate citrate, providing a mechanism to sustain anabolic reactions [32–34] (Fig. 2). Additionally, citrate generated in this way can be turned into malate, which provides another mechanism to generate NADPH via malic enzyme 1 [35, 36]; (Fig. 2).

Cancer cells also exhibit an increased demand for fatty acids, besides glucose and glutamine [37, 38]. Fatty acids can be synthesized endogenously (Fig. 2) or taken up from exogenous sources. In prostate tumors, which import less glucose than other tumors [39], β-oxidation of fatty acids provides an important energy source [40, 41]. Additionally, two recent studies showed that acetate is a bioenergetic substrate for glioblastoma and brain metastases, and it is important for biosynthesis and histone modification in a wide spectrum of tumors [42, 43]. Overall, metabolic phenotypes in cancer cells are plastic, and cancer cells exhibit greater plasticity than normal cells [44].

### Stromal cells and adipocytes can provide building blocks and fuels to tumor cells

Like other aspects of cancer biology, our understanding of tumor metabolism is continuously evolving. Particularly in recent years, some researchers have investigated tumor metabolism in the context of the whole tumor microenvironment. These studies suggest a two-compartment model for understanding tumor metabolism [45–49]. In this model, under the education of cancer cells and inflammatory cytokines, stromal cells and adipocytes become “food donors.” Tumor stromal cells principally include cancer-associated fibroblasts (CAFs), tumor endothelial cells (TECs), and tumor-associated macrophages (TAMs). Catabolism in stromal cells and adipocytes provides fuels and building blocks (see Fig. 2) for the anabolic growth of cancer cells via metabolic coupling [48, 49]. For example, by examining MCF7 breast cancer cells cultured alone or co-cultured with nontransformed fibroblasts, Ko et al. showed that CAFs undergo an autophagic program, leading to the generation and secretion of high glutamine levels into the tumor microenvironment [50]. The glutamine released from CAFs fuel cancer cell mitochondrial activity, driving a vicious cycle of catabolism in the tumor stroma and anabolic tumor cell expansion. Likewise, Nieman et al. showed that triglyceride catabolism in adipocytes drives ovarian cancer metastasis by providing fatty acids as mitochondrial fuels [51]. Furthermore, a study by Sotgia et al. suggested that glycolytic stromal cells produce mitochondrial fuels, L-lactate and ketone bodies, which are transferred to oxidative epithelial cancer cells, driving OXPHOS and mitochondrial metabolism [52]. This is strongly supported by their finding that metastatic breast cancer cells amplify OXPHOS and that adjacent stromal cells are glycolytic and lack detectable mitochondria. In essence, these observations and the two-compartment model are still consistent with Warburg’s original observation that tumors show a metabolic shift towards aerobic glycolysis.

### The metabolic enzymes found to be mutated in tumors include isocitrate dehydrogenase, succinate dehydrogenase, and fumarate hydratase

With the increased interest in tumor metabolism in recent years, mutations in metabolic enzymes have been intensely studied. To date, the metabolic mutations associated with cancer are found mainly in isocitrate dehydrogenase (IDH), succinate dehydrogenase (SDH), and fumarate hydratase (FH). IDH catalyzes the oxidative decarboxylation of isocitrate to produce α-ketoglutarate (α-KG). In humans, there are three different IDH isoforms: IDH1, IDH2 and IDH3 (Fig. 2). IDH1 is located in the cytosol and peroxisomes, while IDH2 and IDH3 are located in mitochondria. IDH1 and IDH2 use NADP^+^ as a cofactor, while IDH3 uses NAD^+^ as a cofactor in the TCA cycle for energy metabolism [53, 54]. All three enzymes convert isocitrate to α-KG.

In 2008, the R132H IDH1 mutation was first found in human glioblastoma multiforme [55]. Subsequently, mutations of the R132 residues were found in leukemic cells of myeloid leukemia (AML) patients [56]. These findings were quickly confirmed by multiple studies involving direct sequencing of IDH1 and its homologue IDH2. Mutations in IDH1 and IDH2 were found in 75 % of grade 2–3 gliomas and secondary glioblastoma, and in about 20 % of AML [57–69]. Additionally, IDH1 and IDH2 mutations were found in several other human tumors, including cartilaginous tumors (75 %), intrahepatic cholangiocarcinoma (10 %), and thyroid carcinomas (16 %) [70–77]. The most common cancer mutations map to single arginine residues in the catalytic pockets: IDH1 (R132) and IDH2 (R172 or R140) [55, 56, 61]. Mutant IDH1/2 forms a dimer with the wild-type protein from the normal allele and displays a neomorphic activity that allows the heterodimeric enzyme to catalyze the reduction of α-KG directly to D-2-hydroxyglutarate (D-2-HG, also known as R-2-HG) in the presence of NADPH [65, 69, 78, 79]. In human glioma with IDH1/2 mutation, the level of D-2-HG accumulates as high as 5–35 mmol L^−1^ [63, 79, 80].

SDH is a highly conserved protein complex with four subunits: SDHA, SDHB, SDHC, and SDHD. SDHA and SDHB are catalytic subunits, and SDHC and SDHD are ubiquinone-binding and membrane-anchorage subunits. SDH functions in the TCA cycle, and as complex II of the electron transport chain (ETC), catalyzes the oxidation of succinate to fumarate in a reaction that generates FADH_2_, and donates electrons to the ETC. Mutations in genes encoding SDH subunits and the SDH assembly factor 2 are found in hereditary paraganglioma and pheochromocytoma, as well as in gastrointestinal stromal tumors, renal tumors, thyroid tumors, testicular seminomas, and neuroblastomas [81]. Over 650 reported cases of SDH mutations have been reported, and these mutations significantly reduce SDH activity. In three cases of paragangliomatosis with SDH mutation, succinate accumulated to a high level of 364–517 μmol g^−1^ protein [82]. Also, Xiao et al. showed that depleting SDH in mice or ectopic expression of tumor-derived SDH mutants resulted in the accumulation of succinate [83].

FH exists as a homotetrameric enzyme that catalyzes the stereospecific and reversible hydration of fumarate to malate. Mutations in the FH gene were first identified in inherited uterine fibroids, skin leiomyoma, and papillary renal cell cancer by a combination of mapping methods [84]. FH mutations were also found in cerebral cavernomas, Leydig cell tumors, and ovarian mucinous cystadenoma with low frequency [85–87]. Over 300 cases of FH mutations have been reported. Like SDH mutations, FH mutations significantly reduce FH activity, resulting in the accumulation of fumarate to a level as high as 417–688 μmol/g protein in hereditary leiomyomatosis and renal cell cancer [82]. The accumulation of fumarate was also observed in cells depleted for *FH* or expressing a tumor-derived FH mutant [83].

The accumulation of D-2-HG, succinate, and fumarate all lead to impaired activity of a class of enzymes called α-KG-dependent dioxygenases. These oxygenases include prolyl hydroxylase (PHD), which causes HIF1α degradation [88]. Hence, the accumulation of D-2-HG, succinate, and fumarate causes HIF1α accumulation. Other α-KG-dependent dioxygenases include the JMJD family KDMs and the TET family of 5mC hydroxylases, which impact epigenetic events [89]. Ultimately, by impacting cellular processes such as hypoxia response and epigenetic modifications, D-2-HG, succinate, and fumarate promote tumorigenesis. Such metabolites whose abnormal accumulation causes both metabolic and nonmetabolic dysregulation and promotes tumorigenesis are often called oncometabolites. However, there is only limited evidence linking these oncometabolites to metastatic progression. For example, treatment with dimethylfumarate, a cell-permeable form of fumarate, strongly reduces invasion and metastasis formation in melanoma [90–92], although overexpression of FH in a FH-deficient renal cell carcinoma line inhibits cellular migration and invasion [93].

### Mitochondrial OXPHOS is essential for ATP generation in most tumor types

As discussed above, mutations in IDH, SDH, and FH may interfere with mitochondrial function and respiration in certain rare tumor types. However, a plethora of studies have shown that mitochondrial function and respiration are critical for many common types of tumors. Over the years, various studies have identified several modes of mitochondrial function in tumorigenesis. For example, mitochondria and cancer are linked through the generation of reactive oxygen species (ROS). Notably, mitochondria generate much of the endogenous cellular ROS through mitochondrial OXPHOS. Under normal physiological conditions, ROS production is highly regulated, at least in part, by complex I [94–98]. When the electron transport chain (ETC) is inhibited by an OXPHOS gene mutation, the ETC electron carriers accumulate excessive electrons, which can be passed directly to O_2_ to generate superoxide anion (O_2_^−^). The O_2_^−^ generated by complex I is released into the mitochondrial matrix and is converted to H_2_O_2_ by the mitochondrial manganese superoxide dismutase (MnSOD). The O_2_^−^ generated from complex III is released into the mitochondrial intermembrane space and is converted to H_2_O_2_ by copper/zinc superoxide dismutase (Cu/ZnSOD). Mitochondrial H_2_O_2_ can then diffuse into other cellular compartments. Mitochondrial ROS are important signaling molecules and potent mitogens [99–101]. Increased production of ROS has long been observed to be a hallmark of many tumors and cancer cell lines [102, 103]. The mechanisms by which ROS promote tumorigenesis have been reviewed extensively elsewhere [95, 104, 105]. Additionally, it is well known that ROS can inhibit tumor progression by inducing apoptosis, and many anti-cancer agents act by generating ROS and inducing cancer cell death [106, 107]. However, this is beyond the scope of this review and is therefore not discussed further here.

Another link of mitochondria to tumorigenesis is OXPHOS. Although it has long been believed that the glycolytic phenotype in cancer is due to defective mitochondrial OXPHOS, as proposed by Otto Warburg [15], this view has been challenged since it was proposed [17]. Many lines of experimental evidence have shown that the function of mitochondrial OXPHOS in most tumors is intact. For example, Guppy and co-workers showed that in the MCF-7 breast cancer cell line, ATP production is 80 % oxidative and 20 % glycolytic [108]. Rodriguez-Enriquez et al. showed that in AS-30D hepatoma tumor cells, cellular ATP is mainly provided by OXPHOS [109]. Furthermore, Rodriguez-Enriquez et al. showed that in both human HeLa and rodent AS-30D fast-growing tumor cells, mitochondria respiration is the predominant source of ATP in both cell types (66–75 %), in spite of an active glycolysis [110]. In glucose-free medium with glutamine, proliferation of both lines is diminished by 30 %, but OXPHOS and the cytosolic ATP level are increased by 50 %. In glutamine-free medium with glucose, proliferation, OXPHOS, and ATP concentration are diminished drastically. In 2004, Zu and Guppy reviewed a plethora of experimental studies regarding glycolytic and oxidative contribution to ATP production in a wide array of tumor cells [111]. Their analyses of previous data showed that the vast majority of tumor cells generate ATP via oxidative phosphorylation.

Notably, a recent study has linked OXPHOS to oncogene ablation-resistant pancreatic cancer cells [112]. Viale et al. showed that a subpopulation of dormant tumor cells surviving oncogene ablation, responsible for tumor relapse, relies on OXPHOS for survival. Furthermore, recent experimental studies have identified transcription factors that promote mitochondrial biogenesis and OXPHOS in cancer cells. For example, LeBleu and co-workers identified the transcription coactivator peroxisome proliferator-activated receptor gamma, coactivator 1alpha (PPARGC1A or PGC-1α) as the transcription factor promoting mitochondrial biogenesis and OXPHOS in cancer cells [113]. They showed that migratory/invasive cancer cells favor mitochondrial respiration and increased ATP production. There is a strong correlation between PGC-1α expression in invasive cancer cells and the formation of distant metastases. In another study, Mauro et al. showed that NF-κB plays a role in metabolic adaptation in cancer by upregulating OXPHOS [114].

### Mitochondrial transfer provides a mechanism for restoring OXPHOS in tumor cells defective in mitochondrial respiration and for promoting tumor progression

Importantly, a recent study involving mtDNA transfer between normal and tumor cells provided further evidence supporting the importance of OXPHOS in cancer progression [115]. Tan et al. showed that tumor cells without mitochondrial DNA (mtDNA) exhibit delayed tumor growth and that tumor formation is associated with the acquisition of mtDNA from host cells [115]. By following mtDNA acquisition in the 4T1 breast carcinoma model, Tan and colleagues found that stable cell lines derived from primary subcutaneous tumors that grew from 4T1ρ^0^ cells showed partial recovery of mitochondrial respiration and an intermediate lag to tumor growth. Cell lines from circulating tumor cells and from lung metastases showed further and staged recovery of mitochondrial respiration, and tumor growth more similar to that of parental 4T1 cells. They demonstrated that restored mitochondrial respiration is critical for the tumorigenic potential of cancer cells without mtDNA [115]. Interestingly, the role of mitochondrial transfer has been observed in canine transmissible venereal tumor (CTVT), which is a highly adapted cancer and is transmitted as an allograft during coition [116]. Rebbeck et al. analyzed mtDNA in 37 transmissible venereal tumors in dogs and comparable mtDNA regions from 15 host animals and 43 published canine mtDNA sequences [117]. Their analyses suggested that these tumors have periodically acquired mitochondria from their hosts, perhaps over a period of 11,000 years when this tumor type originated [116, 117]. It was estimated that the transfer of mitochondria into malignant cells with heavily mutated mtDNA occurs once in about 100 years [117]. Clearly, ample experimental evidence exists to demonstrate the importance of mitochondrial respiration in the progression of many cancers.

### Heme is an essential factor for the proper functioning of OXPHOS complexes and directly regulates many molecular and cellular processes

**Fig. 3:**
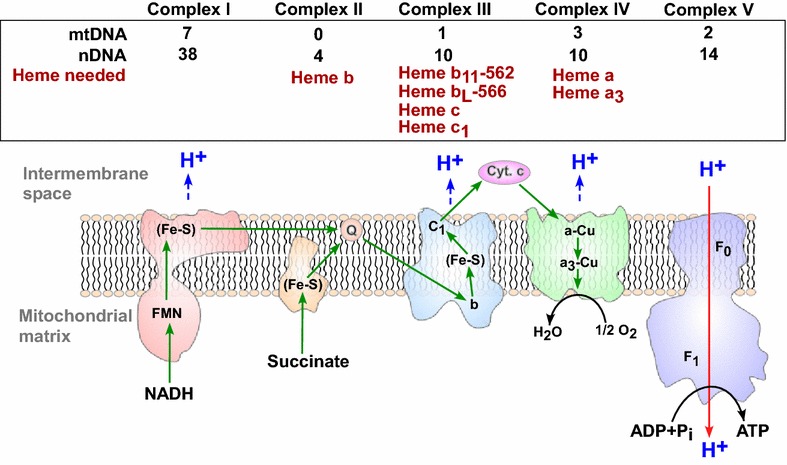
The function and composition of mitochondrial OXPHOS complexes I–V. Shown here are the directions of electron and proton transport by the OXPHOS complexes. Also indicated are the origins of DNA encoding the subunits and the hemes needed for complexes II–IV. *nDNA* nuclear DNA, *mtDNA* mitochondrial DNA

Mitochondrial respiration is carried out by the OXPHOS complexes I–V (Fig. 3) [118]. Complex I, the NADH-coenzyme Q reductase or NADH dehydrogenase, is constituted of 45 polypeptides, of which seven (ND-1, -2, -3, -4, -4L, -5, and -6) are encoded by the mtDNA, and the rest are encoded by nuclear DNA [119, 120]. Complex II, succinate-coenzyme Q reductase or succinate dehydrogenase, contains four nDNA-encoded protein subunits. Complex III, cytochrome bc1 complex or ubiquinol-cytochrome c oxidoreductase, contains 11 subunits, of which one (cytochrome *b*) is encoded by the mtDNA. Complex IV, cytochrome c reductase, is composed of 13 subunits, of which three (CO-I, -II, and -III) are from the mtDNA. Complex V, ATP synthase, contains approximately 16 subunits, of which two (ATP-6 and -8) are from the mtDNA. Complexes I, III, IV, and V retain mtDNA-encoded protein subunits and transport protons (Fig. 3). Importantly, three complexes, II, III, and IV, require heme for proper functioning. Particularly, multiple subunits in complexes III and IV require heme as a prosthetic group, and different forms of heme are present (Fig. 3) [121].

**Fig. 4:**
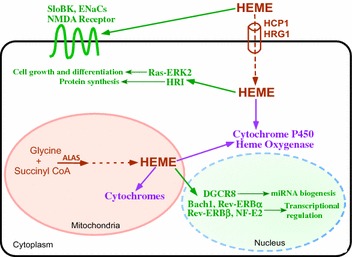
The signaling and structural functions of heme in human cells. Human cells can synthesize heme de novo in mitochondria (the first and rate-limiting enzyme is ALAS, 5-aminolevulinate synthase) or import heme via heme transporters, such as HRG1 and HCP1. Inside cells, heme serves as a prosthetic group in numerous enzymes and proteins that transport, store, or use oxygen, such as mitochondrial cytochromes and cytochrome P450. Additionally, heme directly regulates the activity of diverse cellular signaling and regulatory molecules, such as Bach1, Rev-ERBα, and Rev-ERBβ (transcriptional regulators), as well as DGCR8 (an essential miRNA processing factor) in the nucleus. Heme also regulates HRI (the heme-regulated inhibitor kinase controlling protein synthesis) and the Ras-ERK signaling pathway in the cytoplasm. Furthermore, heme regulates the activity of the NMDA receptor, the SloBK potassium channel, and the ENaCs sodium channel on the cell membrane

The function of heme as a prosthetic group in proteins and enzymes involved in the transport, storage, and utilization of oxygen is well-known [122]. Furthermore, heme directly regulates the expression of proteins and enzymes involved in using oxygen [123]. In humans, heme plays essential roles in many physiological processes, including erythropoiesis, neurogenesis, cell growth and differentiation [123–125]. Heme constitutes 95 % of functional iron in the human body, as well as two-thirds of the average person’s iron intake in developed countries. In the human body, erythroid and hepatic cells use the most heme. Most, if not all, human cells need a basal level of heme for survival. Mammalian cells can synthesize heme endogenously in the mitochondria, or they can import heme from the circulation via heme transporters (Fig. 4) [ [126] and references therein]. In mammalian cells, intracellular heme is used to synthesize various hemoproteins, such as cytochromes, or it can be degraded by heme oxygenase (Fig. 4) [127]. It is also worth noting that heme can serve as a regulatory and signaling molecule and directly regulate transcription, translation, and cell growth and differentiation [125]. For example, in erythroid precursor cells, heme regulates the transcription of globin chains and heme oxygenase genes by modulating the activity of transcriptional regulators, such as NF-E2 and Bach1 [128–131]. Additionally, heme regulates the translation of globin chains by directly controlling the activity of the heme-regulated eIF-2α kinase (HRI) [132, 133]. These mechanisms ensure the coordination of globin chain synthesis with heme synthesis. In neuronal cells, heme can modulate the activity of the NMDA receptor and the Ras-ERK1/2 signaling pathway [134–137]. Furthermore, heme directly regulates the activity of the nuclear receptors REV-ERBα and REV-ERBβ [138, 139], microRNA processing protein DiGeorge critical region-8 (DGCR8) [140], and ion channels (SloBK potassium channel and epithelial sodium channel ENaCs) [141–143], in an array of mammalian cells (Fig. 4).

### Elevated heme flux and function are critical for the proliferation and function of non-small cell lung cancer cells

Interestingly, it has long been observed that inhibiting heme synthesis in various cancer cell lines suppresses cell proliferation and induces apoptosis [144–146]. However, it is not clear how heme deficiency impacts normal cells. This was clarified recently by a study carried out in the authors’ laboratory [147]. In this study, we took advantage of a matched pair of cell lines representing the normal, nonmalignant bronchial epithelial and non-small cell lung cancer (NSCLC) cells developed from the same patient [148, 149]. Using this pair of cell lines and several other NSCLC lines, we examined the differences in bioenergetic activities in normal and cancer cells. We found that the rates of both glucose and oxygen consumption in NSCLC cells are elevated, with the elevation of oxygen consumption greater than glucose consumption [147]. Next, we showed that the rate of heme synthesis is increased significantly in the NSCLC cells, compared to the normal lung cells. Additionally, we showed that the expression level of the rate-limiting heme synthetic enzyme, ALAS1, is highly elevated in NSCLC cells and tumors. Further, the levels of two heme transporters HCP1 and HRG1 [150, 151] are dramatically increased in NSCLC cells and tumors, compared to the normal cells [147]. The increased availability of heme is expected to intensify the production of oxygen-utilizing hemoproteins. Indeed, we found that the levels of cytoglobin, cytochrome c, cytochrome P450 CYP1B1, and Cox-2 are significantly increased in NSCLC cells and tumors [147]. Our results revealed that both heme biosynthesis and uptake are intensified to enhance heme availability for the production of oxygen-utilizing hemoproteins in cancer cells and xenograft tumors [147].

Increased levels of heme and oxygen-utilizing hemoproteins presumably contribute to intensified oxygen consumption in cancer cells. Conversely, depleting heme in cancer cells is expected to cause a lack of hemoproteins, leading to reduced oxygen consumption and cellular energy generation. Indeed, we found that oxygen consumption in the NSCLC cells is selectively reduced when cells are cultured in heme-depleted medium [147]. In contrast, heme depletion in the medium does not affect oxygen consumption in normal cells. Further, we showed that lowering heme levels strongly suppresses NSCLC cell proliferation, colony formation, and migration [147]. Together, our results showed that heme availability is significantly increased in cancer cells and tumors, which leads to elevated production of hemoproteins, resulting in intensified oxygen consumption and cellular energy production for fueling cancer cell progression [147].

The selective importance of heme in tumor cell proliferation and function is also consistent with the previous observation that NSCLC cells require serum (containing heme) for maintenance and proliferation, whereas the normal lung cells survive and proliferate better with growth factors in the absence of serum [148, 149]. Further, the preferential requirement of NSCLC cells for heme is in complete agreement with the critical roles of heme in mitochondrial respiratory chain complexes. As shown in Fig. 4, OXPHOS complexes II, III, and IV all require heme for proper functioning. By logical reasoning, tumor cells that depend mainly on OXPHOS for ATP generation should require elevated levels of heme and hemoproteins for proliferation and function.

### Clonal evolution and genetic heterogeneity likely contribute to the remarkable versatility of tumor cells in the use of bioenergetic substrates

In recent years, whole-genome and whole-exome sequencing studies have provided an ever-expanding survey of somatic aberrations in cancers [152–156]. Such large-scale sequencing studies have revealed a high degree of genetic heterogeneity among patients with the same type of cancer, namely inter-tumor heterogeneity, and that within a single tumor or sample, namely intra-tumor heterogeneity [157–165]. For example, Gerlinger et al. found that over half the mutations in primary tumor and its various metastases of the same advanced renal cell carcinoma are different [166]. Likewise, several groups have demonstrated the vast heterogeneous mutational landscape of pancreatic cancer by analyzing data from whole-genome and whole-exome sequencing [167–169]. Additionally, Ellsworth et al. found genomic heterogeneity within primary breast carcinomas and among regional LN metastases [170]. They concluded that metastasis is a complex process influenced by primary tumor heterogeneity and variability in the timing of dissemination. Furthermore, Leiserson et al. performed a pan-cancer analysis of mutated networks in 3281 samples from 12 cancer types from the Cancer Genome Atlas (TCGA) [171]. They identified 16 significantly mutated subnetworks that comprise well-known cancer signaling pathways as well as subnetworks with less characterized roles in cancer, including cohesin, condensin, and others. In a comprehensive review, Vogelstein et al. summarized the genes altered in a high percentage of tumors and a much larger number of genes altered infrequently [163]. They reported ~140 driver genes whose intragenic mutations can promote or drive tumorigenesis. These driver genes can be classified into 12 signaling pathways that regulate three core cellular processes: cell fate, cell survival, and genome maintenance.

Data from these large-scale cancer genome sequencing studies also support clonal evolution as the mechanism responsible for generating intra-tumor heterogeneity (ITH). Clonal evolution was initially proposed by Nowell [172]. It refers to the process in which cancer cells accumulate genetic and epigenetic changes over time, giving rise to new subclones. It suggests that cancer evolves by a process of clonal expansion, diversification, and selection within the tissue ecosystems. Clonal evolution can be linear evolution or branched evolution [158, 159]. Evidence of clonal evolution is found in many tumors. For example, evaluation of genomic heterogeneity within primary breast carcinomas and among axillary LN metastases indicated that multiple clonal cell lineages exist in every primary tumor and between many metastatic deposits from the same patient [170]. Two recent studies revealed substantial intra-tumoral heterogeneity within lung adenocarcinomas [173, 174]. Cancer evolution and tumor heterogeneity likely contribute to tumor recurrence and the emergence of drug-resistant disease [175–177]. Under therapeutic pressure, those tumor clones that are most adaptive or resistant to treatment will be selected. These clones will then dominate and populate the tumor rendering it highly resistant to the given therapy. Further, some of these resistance pathways lead to multidrug resistance, generating an even more difficult clinical problem to overcome. Likewise, high mutational heterogeneity and subclonal mutation fraction can lead to increased likelihood of tumor recurrence.

Very likely, changes in tumor cell bioenergetic characteristics accompany tumor progression, recurrence, and drug resistance. Tumor cells are remarkably versatile in their ability to adapt to and take advantage of the environment to support their proliferation and function. Firstly, tumor cells use a variety of fuels, including glucose, glutamine, fatty acids, ketone bodies, and acetate [37–43]. Secondly, tumor cells from the same type of tumors can exhibit great variations in metabolic and bioenergetic phenotypes. Notably, different NSCLC cell lines exhibit a wide range of dependence on glutamine [30]. These cell lines also show a varying degree of increased oxygen consumption rates, as well as heme synthesis and uptake rates. Evidently, tumor cells adapt to the environment and adopt specific bioenergetic features in order to take advantage of whatever fuels are available. For example, tumor cells in an environment rich in adipocytes would likely adapt to preferentially use fatty acids, while tumor cells in an environment rich in myocytes may adapt to preferentially use glutamine. Clonal evolution enables different tumor cells to adopt metabolic and bioenergetic phenotypes fit to their environment. Such variations in tumor bioenergetic characteristics are likely underpinned by genetic heterogeneity. That is, the aforementioned diverse mutations in signaling pathways and networks would ultimately impact the expression and activity of metabolic enzymes, thereby enabling tumor cells to adopt specific bioenergetic features fit for their unique environment.

## Conclusions

Recent advances in cancer research have clarified many issues relating to tumor bioenergetics. Some important points include the following: (1) High glycolytic rates in tumors and mitochondrial respiration often operate simultaneously in tumors. Increased glycolysis most likely contributes building blocks for biosynthesis. (2) Glutamine is the preferred oxidative fuel for tumor cells. (3) Tumor cells can use a range of fuels including glucose, glutamine, fatty acids, and acetate. (4) Mutations in metabolic enzymes are found mainly in three enzymes involved in the TCA cycle. (5) Mitochondrial respiration can be restored by mitochondrial transfer in tumor cells defective in OXPHOS, and it is critical for the initiation and metastasis of diverse tumors. (6) Elevated heme flux and function lead to intensified oxygen consumption in NSCLC cells, fueling cancer cell proliferation, migration, and colony formation. (7) Lowered heme availability selectively diminishes the proliferation and function of NSCLC cells. (8) Clonal evolution contributes to a high degree of genetic heterogeneity in tumors, which likely underpin metabolic and bioenergetic versatility of tumor cells, as well as tumor recurrence and drug resistance. Evidently, clonal evolution likely enables NSCLC cells to enhance heme synthesis and uptake, in order to increase their cellular energy generation. Heme coordinates the production and function of OXPHOS complexes. Hence, increasing heme availability provides an effective way to upregulate OXPHOS complexes and mitochondrial respiration for energy generation. It is likely that this mechanism involving elevated heme flux and function operates in other types of tumors besides NSCLC tumors to promote tumor development and progression.

Recent research has also provided ample evidence showing that many types of tumors indeed consume larger amounts of glucose, compared to normal tissues, as Warburg originally observed [15]. However, his hypothesis that tumor mitochondria have impaired respiration is largely incorrect for most types of tumors, as discussed extensively in this review. The observed large increases in glucose consumption in tumor tissues can be attributed to increased demand for building blocks in tumor cells and to increased glucose consumption in stromal cells, which in turn provide oxidative fuels, such as lactate, to tumor cells. Nonetheless, Warburg’s original observation has motivated generations of scientists to better understand tumor bioenergetics, and this will undoubtedly lead to a more holistic approach in cancer research and therapeutics.

## Abbreviations

ALAS1: 5′-aminolevulinate synthase 1
ETC: electron transport chain
HCP1: heme carrier protein 1
HIF1α: hypoxia-inducible factor 1α
HRG1: heme-responsive gene 1
HRI: heme-regulated eIF-2α kinase
IDH: isocitrate dehydrogenase
NMDA: N-methyl-D-aspartate
NSCLC: non-small cell lung cancer
OXPHOS: oxidative phosphorylation
ROS: reactive oxygen species
SDH: succinate dehydrogenase
