# Correction to: A holistic view of cancer bioenergetics: mitochondrial function and respiration play fundamental roles in the development and progression of diverse tumors

**DOI:** 10.1186/s40169-018-0186-5

**Published:** 2018-03-02

**Authors:** Md Maksudul Alam, Sneha Lal, Keely E. FitzGerald, Li Zhang

**Affiliations:** Department of Biological Sciences, University of Texas at Dallas, Mail Stop RL11, 800 W, Campbell Road, Richardson, TX 75080 USA

## Correction to: Clin Trans Med (2016) 5:3 10.1186/s40169-016-0082-9

In this Correction, the authors would like to acknowledge that the original publication of the article “*A holistic view of cancer bioenergetics: mitochondrial function and respiration play fundamental roles in the development and progression of diverse tumors*” [1] was supported by CPRIT (Cancer Prevention & Research institute of Texas) Grant RP160617.
